# Novel viruses in salivary glands of mosquitoes from sylvatic Cerrado, Midwestern Brazil

**DOI:** 10.1371/journal.pone.0187429

**Published:** 2017-11-08

**Authors:** Andressa Zelenski de Lara Pinto, Michellen Santos de Carvalho, Fernando Lucas de Melo, Ana Lúcia Maria Ribeiro, Bergmann Morais Ribeiro, Renata Dezengrini Slhessarenko

**Affiliations:** 1 Programa de Pós-Graduação em Ciências da Saúde, Faculdade de Medicina, Universidade Federal de Mato Grosso, Cuiabá, Mato Grosso, Brazil; 2 Departamento de Biologia Celular, Instituto de Ciências Biológicas, Universidade de Brasília, Brasília, Distrito Federal, Brazil; 3 Departamento de Biologia e Zoologia, Instituto de Biociências, Universidade Federal de Mato Grosso, Cuiabá, Mato Grosso, Brazil; Metabiota, UNITED STATES

## Abstract

Viruses may represent the most diverse microorganisms on Earth. Novel viruses and variants continue to emerge. Mosquitoes are the most dangerous animals to humankind. This study aimed at identifying viral RNA diversity in salivary glands of mosquitoes captured in a sylvatic area of Cerrado at the Chapada dos Guimarães National Park, Mato Grosso, Brazil. In total, 66 Culicinae mosquitoes belonging to 16 species comprised 9 pools, subjected to viral RNA extraction, double-strand cDNA synthesis, random amplification and high-throughput sequencing, revealing the presence of seven insect-specific viruses, six of which represent new species of *Rhabdoviridae* (Lobeira virus), *Chuviridae* (Cumbaru and Croada viruses), *Totiviridae* (Murici virus) and *Partitiviridae* (Araticum and Angico viruses). In addition, two mosquito pools presented Kaiowa virus sequences that had already been reported in South Pantanal, Brazil. These findings amplify the understanding of viral diversity in wild-type Culicinae. Insect-specific viruses may present a broader diversity than previously imagined and future studies may address their possible role in mosquito vector competence.

## Introduction

Viruses may represent the most abundant and diverse microbes on Earth [1–3]. Previously unrecognized virus species and variants continually emerge, favored by globalization, climate changes, viral RNA plasticity with adaptation to vectors and hosts, ecotourism, uncontrolled urbanization and proximity among urban centers and sylvatic areas, posing a significant global health concern, especially in developing tropical regions [4–6]. The research of new species is challenging for traditional and current detection methods due to viral profusion [7]. High-throughput sequencing (HTS) lead to the identification of previous uncharacterized viruses, virulence factors and more accurate and complete viral genomic data. Thus, enlightening viral ecology, diversity and evolution [3,8].

The interest on new human, animal and plant viruses naturally drew research efforts to metagenomic studies involving invertebrates. At least 220 viruses are recognized human pathogens [9], 150 of which are transmitted by arthropods [10], classified as arthropod-borne viruses or arboviruses [11]. Mosquitoes are the most important vectors of arboviral diseases to humans [12], and are considered one of the deadliest animals by the World Health Organization [13]. Arboviruses are originally maintained in nature by enzootic cycles of transmission [5]. A high density of competent vectors and susceptible amplifier hosts, mainly birds, primates and small mammals is a fundamental condition for maintenance of arboviruses [5,14].

For a mosquito to become competent for arbovirus transmission, a complex of multifactorial physical barriers and evolutive selections must be overcome by the virus, until a persistent infection is established in their salivary glands, secreting large amounts of viral particles in their saliva [15].

Metagenomic studies involving insects surprisingly revealed a higher genetic biodiversity than observed in viruses affecting vertebrates [8,16,17], suggesting that most viral infections in arthropods are asymptomatic or latent [7].

Insect-specific viruses (ISV) only replicate in invertebrate cell lines and can interfere with the replication of some arboviruses in mosquito cells, probably altering vector competence [18–20]. Most ISV are classified in the same taxons and genera of arboviruses, such as the *Flaviviridae*, *Rhabdoviridae*, *Togaviridae*, *Bunyaviridae* and *Reoviridae* families, as well as the *Mesoniviridae*, *Tymoviridae*, *Birnaviridae*, *Totiviridae*, *Partitiviridae*, *Chuviridae* families and in the negevirus taxon [21].

This study aimed to investigate the diversity of viral RNA genomes in salivary glands of mosquitoes captured in a protected Cerrado area comprising the Chapada dos Guimarães National Park (CGNP), State of Mato Grosso (MT). Cerrado, a tropical savannah that originally covered 22% of the Brazilian territory, is considered the second greatest phytogeographic domain in South America and one of the 34 hotspots of global biodiversity [22–24].

## Materials and methods

### Study area

**Fig 1:**
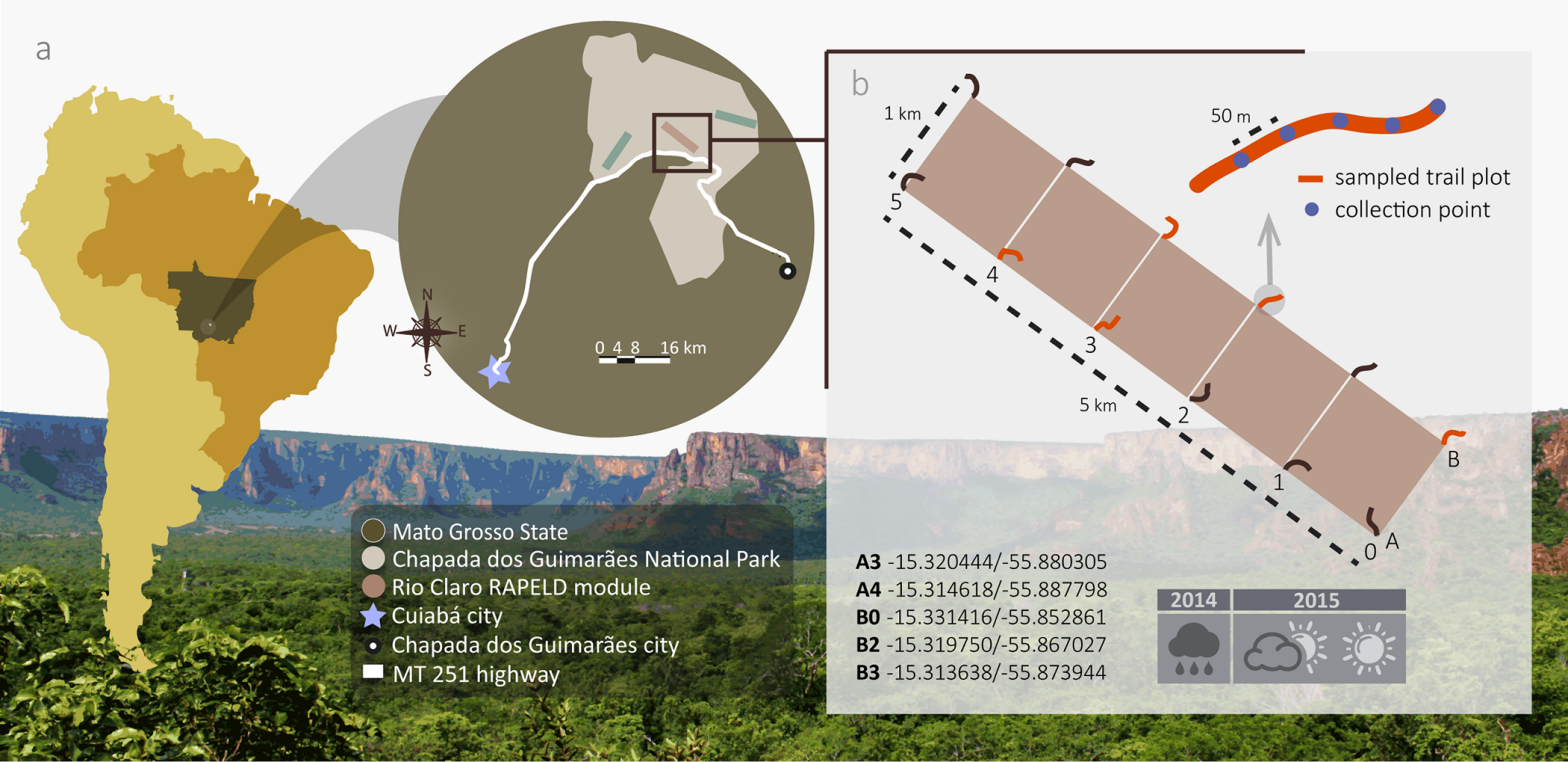
Mosquito collection points location in different climatic periods between 2014–2015 at Chapada dos Guimarães National Park (CGNP). (**a**) CGNP location in State of Mato Grosso, Central-Western Brazil, containing three Rapid Assessment Surveys for Long-Term Ecological Research modules (RAPELD) (green and brown rectangles). (**b**) Rio Claro RAPELD module schematic representation, indicating the sampled plots and their trails in red (A3, A4, B0, B2 and B4 with their respective geographical coordinates). Blue dots represent the collection points within each trail in the enlarged view.

CGNP is a protected sylvatic area of Cerrado with 326,30 km^2^ and intense eco-touristic activity, located in the South-Central region of MT, Midwestern Brazil, in close proximity to urban centers (35 km from Cuiabá, capital of the State) (Fig 1A). This region presents altitudes ranging between 200 and 900 m and tropical climate with a mean temperature of 25°C, 1,900 mm annual rainfall and two well-defined seasons: a rainy summer (October-March) and a dry winter (April-September) [25].

### Mosquito sampling

Collections were carried out in five plots of the Rio Claro RAPELD (Rapid Assessment surveys for Long-Term Ecological Research) module [26] present in the CGNP. The module covers an area of 5 km^2^ subdivided into 12 equidistant plots, each with 250 m of topographical isocline that works as a sampling trail. The choice was based on proximity to water collections, riparian vegetation, bird landing spots and easier access to vehicle (Fig 1B).

Adult Culicinae mosquitoes were captured for two consecutive days with Nasci aspirators (1 pm to 8 pm) and CDC light traps (6 pm to 6 am) in December 2014 and April and September 2015, characterizing rainy, transition and dry periods, respectively. Nasci aspirator catches were carried out for 30 min in each plot sampling trail, and CDC light traps were installed every 50 mat a height of 1.5 m above ground level. Collections were performed in accordance with Brazilian laws, approved by SISBIO/Ministry of the Environment, license number 43909–1.

Specimens were maintained with artificial feeding (sugar solution 20%) under controlled temperature and humidity for 3–4 days until the identification with taxonomic keys in a dormant state [27]. Females were pooled into 1–20 individuals by genus and collection season, followed by salivary glands dissection [28] in phosphate buffer and stored at -80°C (Table 1).

**Table 1:** Pools of Culicinae specimens captured in the Rio Claro RAPELD module at Chapada dos Guimarães National Park, Mato Grosso, Brazil.

Pool	Species [n specimens]	Period*	Plots	RNA	DNA product	Total reads (nt)
M01	*Psorophora albigenu* [2]	Rainy	A3	10	7.649	20,091,498
	*Psorophora ciliata* [1]		A3			
	*Psorophora cingulata* [3]		A3			
	*Psorophora ferox* [5]		A3			
	*Psorophora lanei* [1]		B2			
	*Psorophora lineata* [1]		B2			
	*Psorophora longipalpus/albipes* [1]		B2			
M02	*Haemagogus janthinomys* [4]	Rainy	A3, B2	6.3	36.217	3,978,638
M03	*Stegomyia albopicta* [1]	Rainy	A3	6.2	8.800	11,717,278
	*Ochlerotatus* sp. [7]		A3, B2, B3			
M04	*Ochlerotatus serratus* [1]	Transitional	A3	4.8	4.356	12,032,638
	*Ochlerotatus crinifer* [1]		A3			
M05	*Mansonia wilsoni* [3]	Transitional	A3, B3	5.6	25.607	16,471,976
M06	*Culex* sp. [12]	Transitional	A3	9.2	37.209	5,683,104
M07	*Psorophora dimidiata* [2]	Transitional	A4,	9	38.244	7.839,992
	*Psorophora pseudomelanota* [1]		B0, A3			
M08	*Stegomyia albopicta* [3]	Transitional	B0, B3	11.5	46.529	8,932,600
M09	*Wyeomyia* sp. [17]	Dry	A3, B0	8.3	127.090	11,941,868

*Climatic period. Rainy: December, 2014; Transitional: April, 2015; Dry: September, 2015. n: number

RNA and DNA concentration is presented in ng/μL.

### Viral RNA extraction, reverse transcription and dscDNA synthesis

Viral RNA was extracted from 200 μL of minced salivary glands using High Pure Viral RNA Kit (Roche, USA), without carrier RNA. RNA was quantified (quantifluor RNA system, Promega) and reverse transcribed in random reactions with 20 μL final volume using 20–957 ng of RNA, 5 μM of K-random-S primer [29], 0.25 mM dNTP mix, buffer, 5 mM of MgCl_2_, 16 U of RNAse out (Invitrogen, USA) and 100 U of Go Script Reverse Transcriptase (Promega, USA) at 25°C for 5 min and 42°C for 60 min. The second strand of cDNA (dscDNA) was synthesized using 20 μL of cDNA, 2 μM of the same random primer, buffer, 0.2 mM of dNTP mix and 5 U of DNA Pol I Large Klenow Fragment (Promega, USA) in 25 μL final volume, incubated at 25°C for 20 min and 75°C for 20 min.

### Viral random PCR

Samples were amplified in quintuplicate using 5 μL of dscDNA, 2 μM of K-S primer [29], 2.5 U of GoTaq Hot Start Polymerase (Promega, USA), Buffer, 2mM MgCl_2_, 0.2 mM of dNTP mix and ultrapure water in 50 μL final volume and amplified as described by Kluge et al. [30]. Final product was purified with polyethylene glycol 8000 20%, eluted in 50 μL of ultrapure RNAse free water and quantified using the quantifluor one dsDNA system (Promega).

### High-throughput sequencing and analysis

cDNA libraries were constructed using Illumina TruSeq RNA v2 Kit. Samples were sequenced using 2 x 100 paired-end reads in two lanes with 60 GB on a HiSeq 2500 platform (Illumina, USA) at Macrogen (Seoul, Korea).

Sequence read data were quality checked using FastQC (v0.11.5) and trimmed to remove terminal low-quality, Illumina adapters and random primer adaptor using Trimmomatic (v0.36), filtering out reads shorter than 60 bases (parameters: ILLUMINACLIP: TruSeq3-PE.fa:2:20:10, LEADING: 3, TRAILING: 3, SLIDINGWINDOW: 4:30, MINLEN: 60). These reads were assembled using the CLC Genome Workbench (v6.5.2) and Velvet (v2.1.10) with various kmer size parameters (25, 40, 60 and 90). Resulting contiguous sequences (contigs) were used to search against the viral RefSeq database by BLASTx tool and those with viral hits were searched against the non-redundant sequence database (nr) using BLASTx to confirm the viral identity. Only those hits with e-values of less than 1e-3 were used.

To further extend the viral contigs, the reads were mapped back to the viral contig and the resulting contig was used as seeds for another attempted assembly until genome completion or no further extension. Contig mapping and genome annotation were performed using Geneious (v9.1.7). The on-line open access software TMHMM (v2.0) (http://www.cbs.dtu.dk/services/TMHMM/) was used to predict the transmembrane domains. All the sequences obtained in this study were deposited in GenBank (NCBI; Table 2).

**Table 2:** Viral sequences obtained from the salivary glands of Culicinae mosquitoes captured in the Rio Claro module, Chapada dos Guimarães National Park, Mato Grosso, Brazil.

**Pool**	**GenBank**	**Virus**	**Best hit**	**Length (nt)**	**aa**	**Query cover**	**E-value**	**Classification**	**Hits Genome**
	accession				identity	(%)			
	number				(%)				
**M03**	MF344589	Kaiowa virus	putative glycoprotein	705	100	68	3e-94	*Chuviridae*	ssRNA-
		BR/MT-M03	[Kaiowa virus] ANW72242						
**M05**	MF344587	Murici vírus	RdRp	903	41	99	2e-69	*Totiviridae*	dsRNA
			[Anopheles totivirus]						
			AOR51364						
**M05**	MF344596	Cumbaru virus	putative glycoprotein	472	69	93	1e-77	*Chuviridae*	ssRNA-
			[Kaiowa virus] ANW72242						
**M05**	MF344586	Araticum virus	RdRp	1348	56	56	3e-168	*Partitiviridae*	dsRNA
			[Hubei partiti like vírus 42]						
			APG78281						
**M06**	MF344585	Angico virus	RdRp	1143	57	57	4e-155	*Partitiviridae*	dsRNA
			[Hubei partiti-like virus 48]						
			APG78218						
**M07**	MF344588	Croada virus	putative glycoprotein	558	72	76	8e-72	*Chuviridae*	ssRNA-
			[Kaiowa virus] ANW72242						
**M08**	MF344590	Kaiowa virus BR/MT-M08	putative glycoprotein	1353	99	67	0.0	*Chuviridae*	ssRNA-
			[Kaiowa virus] ANW72242						
**M08**	MF344591	Lobeira virus (nucleoprotein)	Nucleoprotein	1219	49	88	7e-116	*Rhabdoviridae*	ssRNA-
			[North Creek virus]						
			AGY80340						
**M08**	MF344592	Lobeira virus	Phosphoprotein	515	42%	40%	3e-32	*Rhabdoviridae*	ssRNA-
			[Riverside virus 1]						
			AMJ52361*						
**M08**	MF344593	Lobeira virus	Matrix protein	545	42%	40%	3e-32	*Rhabdoviridae*	ssRNA-
			[Riverside virus 1]						
			AMJ52361*						
**M08**	MF344594	Lobeira virus	Glycoprotein [Riverside virus 1] AMJ52367	1620	29%	46%	2e-14	*Rhabdoviridae*	ssRNA-
**M08**	MF344595	Lobeira virus	Large protein	8875	58%	93%	0.0	*Rhabdoviridae*	ssRNA-
			[Riverside virus 1]						
			AMJ52368						

aa: amino-acid; BR: Brazil; MT: Mato Grosso; RdRp: RNA dependent RNA polymerase

* The presented results correspond to the concatenated genes (phosphoprotein plus matrix protein).

### Inoculation in cell culture and RT-PCR for a rhabdovirus

The salivary glands supernatant of the pool positive for Lobeira virus was inoculated into C6/36 cells (1:10 dilution) cultivated in L-15 medium supplemented with 5% fetal bovine serum and incubated at 28°C with 5% CO_2_, monitored for 7 days for cytophatic effect identification.

The supernatant was stored at -80°C and an aliquot subjected to RNA extraction, reverse transcription with primers designed using Geneious for a region between N and P genes (NPLOBF-AGTGGGAGTGGTTCAGACTG; NPLOBR-AAGTGTCTTCTAGATCCCGGT at 1 μM; 500 bp), a region of G gene (GLOBF-GTGAACGTCGTATAGTGAAATCCG; GLOBR-GCACCCCATCCTTCAAAATGA at 1 μM; 250 bp) and a region of L gene (LLOBF-AGCAGGTGGATTAGAGGGGC; LLOBR- ATATCCGCTGCCTGAAGAGTC at 1 μM; 600 bp).

PCR reactions included cDNA (7 μL), buffer, MgCl_2_ (2 μM), dNTP mix (0.2 μM), ultrapure water and 2.5 U of HotStart DNA polymerase (Promega, USA) and the same forward and reverse primers used in reverse transcription. These reactions were amplified at 94°C for 2 min, 30 cycles of 94°C for 1 min, 57°C for 1 min and 72°C for 1 min, and a final extension of 72°C for 5 min. DNA products were identified in 1.5% agarose gels after eletrophoresis.

### Phylogeny

Potential viral proteins identified in this study were used to query NCBI nr protein database using the BLASTp tool to determine the closest relative sequences, its taxonomic classification and similarity. Then, these sequences were aligned with their corresponding homologs and related taxonomic reference sequences using MAFFT software (v7.221). The best evolutionary model was determined by the ProtTest server (2.4) (http://darwin.uvigo.es/software/prottest2_server.html/) [31] for each alignment. The evolutionary history was inferred by maximum likelihood method (ML) based on the Le_Gascuel_2008 model. A discrete gamma distribution was used to model the evolutionary rate differences among the sites (four categories). Evolutionary analyses were conducted using MEGA7. Phylogenies were edited with FigTree v1.4.3 (http://tree.bio.ed.ac.uk/software/figtree/) [32].

## Results

### Sequencing analysis

Illumina sequencing yielded 98,689,592 reads from nine pools comprising 66 adult mosquitoes, reduced to 32,926,122 reads with a median length of 101 nt after trimming. These data generated 129,321 contigs varying from 117 to 2628 nt. Viral RefSeq BLASTx revealed 1050 viral hits (0.81%). BLASTx nr selected 47 contigs (4.47%) as potentially belonging to viruses, classifying the remainder as probable sequences of insects (524, 49.90%), bacteria (242, 23.04%), fungi (111, 10.57%), vertebrates (31, 2.95%) and others taxons (95, 9.04%).

After de novo assembly, 11 virus-like sequences were obtained from five mosquito salivary gland pools, indicating the presence of seven different viruses between each other and previously known viruses. Of these contigs, nine translated sequences showed ≤ 75% amino acid (aa) identity to unclassified viruses related to *Rhabdoviridae*, *Totiviridae*, *Partitiviridae* and the recently classified *Chuviridae* family. These sequences represent six new viruses different between each other, which were named using popular names of typical trees found in Cerrado biome. In addition, two sequences yielded 99.5% similarity among themselves and ≥ 99% identity with sequences of the putative glycoprotein of Kaiowa virus (KAIV), originally described in *Culex* spp. [33], indicating the detection of a strain of this virus in the salivary glands of a different species, *Stegomiya albopicta* (Table 2).

### Rhabdoviridae

**Fig 2:**
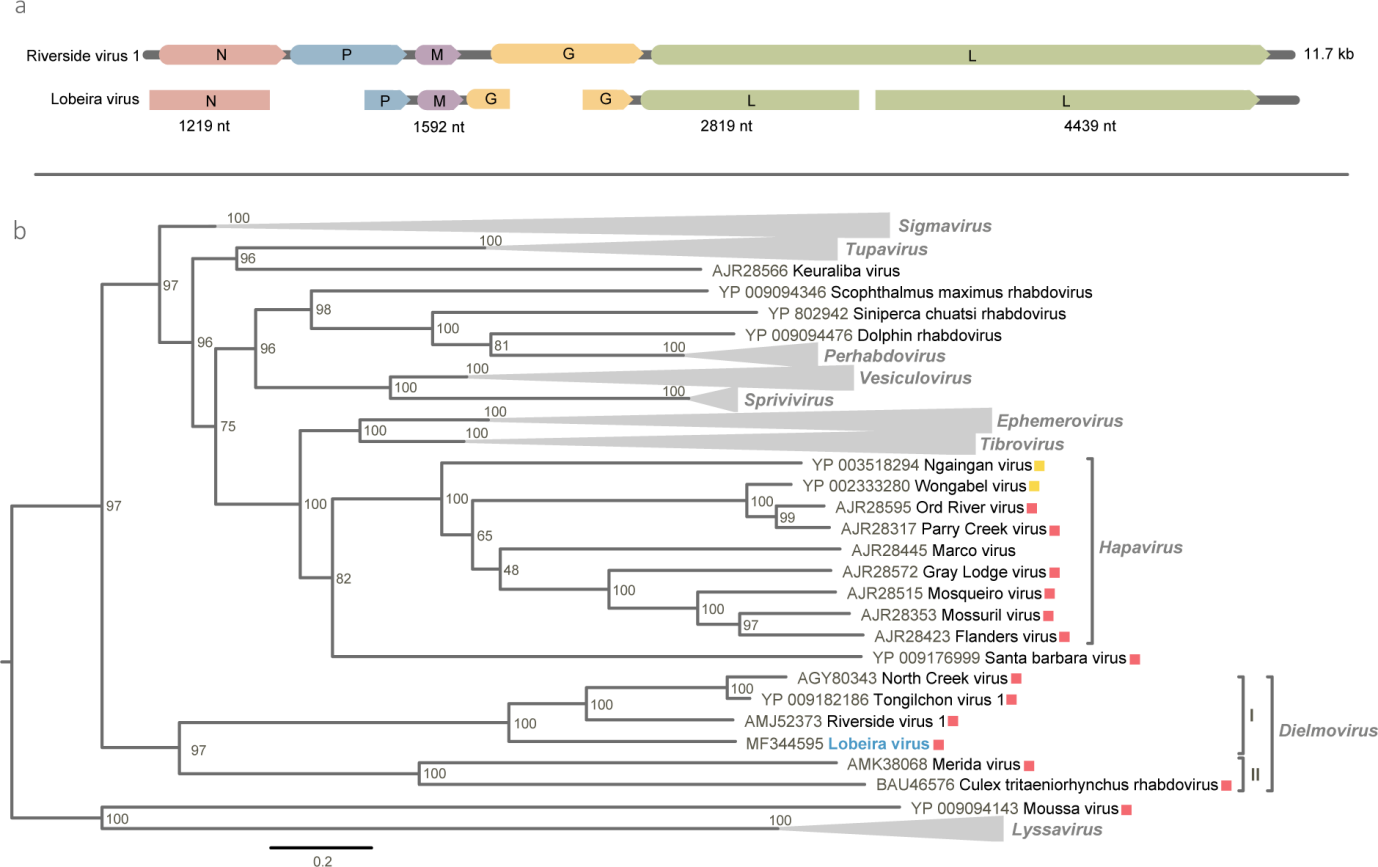
Lobeira virus genome map and phylogeny. (**a**) Genomic organization of Lobeira virus and structure-based alignment with Riverside virus 1. (**b**) Maximum likelihood phylogenetic tree for Large protein of Lobeira virus (in blue) with dimarhabdovirus supergroup members and selected rhabdovirus-like sequences related to Lobeira virus by BLASTp search. Phylogeny was rooted on the branch of *Lyssavirus* genus. Viruses originally found in mosquitoes and other arthropods are marked in red and yellow, respectively. Bar indicates amino acid substitutions per site.

In one pool containing three specimens of *Stegomyia albopicta* (M08) four viral sequences were identified during the BLASTp search. These belong to the same virus and are most closely related to North Creek rhabdovirus (NOCRV) and Riverside virus 1 (RISV), which were discovered infecting *Culex sitiens* [34] and *Ochlerotatus* mosquitoes [35] The closest match for these contigs were the genes of the nucleocapsid protein (N) of NOCRV, the matrix protein (M)t and two different regions of the large protein (L) gene of RISV. The two regions of L protein were concatenated based on alignment with the L protein sequences of RISV, NOCRV and Tongilchon virus 1. These partial genomic sequences belong to the same novel virus, which we named Lobeira virus (LOBV) (Table 2). According to our phylogenetic tree based on L protein, this virus clustered with high node values with RISV, NOCRV and Tongilchon virus 1 forming clade I of the recently proposed *Dielmovirus* genus, dimarhabdovirus supergroup (dipteran-mammal-associated rhabdovirus) [36]. Together with clade II, clade I behaves as a basally rooted lineage of dimarhabdovirus supergroup (Fig 2). Lobeira virus was isolated in c6/36 cells, revealing rounded and dead cells in the supernatant.Isolation was confirmed by three RT-PCR protocols for different genomic regions of Lobeira virus.

### Chuviridae

**Fig 3:**
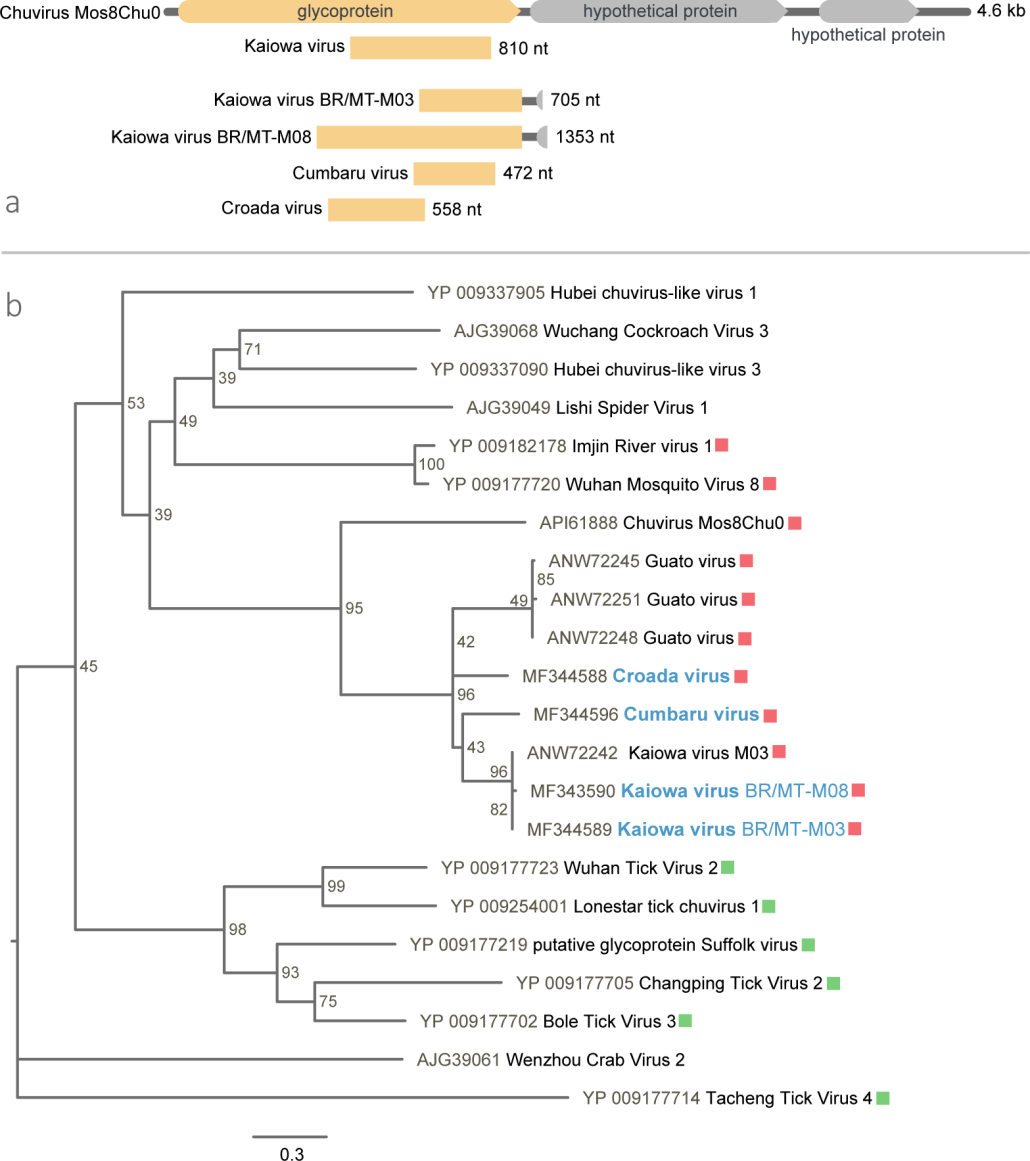
Croada, Cumbaru and Kaiowa viruses partial genomic maps and phylogeny. (**a**) Schematic representation of structure-based alignment of Kaiowa virus BR/MT-M03 and BR/MT-M08, Croada virus, Cumbaru virus and Chuvirus Mos8Chu0. (**b**) Maximum likelihood phylogenetic tree for the glycoprotein of Kaiowa, Croada and Cumbaru viruses (marked in blue) with members of *Chuviridae* family. Viruses originally found in mosquitoes and ticks are marked in red and green, respectively. Bar indicates amino acid substitutions per site.

Four chuvirus partial glycoprotein sequences were detected in different pools (Table 2). Two of these, found in a *St*. *albopicta*-only and *St*.*albopicta* and *Ochlerotatus* pools, correspond to the putative glycoprotein of KAIV, presenting 99 and 100% similarity with the original KAIV sequence [33]. The KAIV sequence found in the M08 pool (BR/MT-M08 KAIV) codes for a 399-aa polypeptide, representing an increase of 129 aas in the original KAIV glycoprotein ends, which is differentiated by only one base pair (bp), culminating in the exchange of a leucine for a proline. According to the BLASTp matches, the BR/MT-M08 KAIV is mostly related to Guato virus (GUTV) and to *Chuviridae* viruses, such as Chuvirus Mos8Chu, Imjin River virus 1 and Wuhan mosquito virus 8, but with reduced aa identity (≅30%). Both KAIV sequences encode the end of a putatitve glycoprotein ORF, with a poly A tail at the 3’UTR and the beginning of a second ORF (Fig 3A).

Two other chuvirus sequences were found in the salivary glands of *Mansonia wilsoni* (M05) and *Psorophora* (M07) mosquitoes, coding for 157 and 186 aas. These contigs showed the highest aa identity (69 and 72%, respectively) with the KAIV glycoprotein sequence by BLASTp search (Table 2). Therefore, owing to the relatively low aa identity found, these sequences belong to two new viruses different from each other, named Cumbaru virus (CUMV) and Croada virus (CROV). CUMV sequence presents a transmembrane domain between 92 and 114 aa position, indicating that this is probably a viral envelope glycoprotein.

The ML phylogenetic tree for KAIVs, CUMV, and CROV included the representative *Chuviridae* viruses and the most closely related chuvirus species. CUMV, CROV, KAIV and GUTV clustered into a distinct lineage to Chuvirus Mos8Chu0, inserted in a major group with other viruses originally described in insects, dismembered from tick viruses (Fig 3).

### Totiviridae

A sequence with 903 nt encoding part of the putative RNA dependent RNA polymerase (RdRp) gene was found in the *Ma*. *wilsoni* pool (M05). This sequence showed the highest aa identity (≤ 41%) with the Anopheles totivirus (AToV), identified in *Anopheles gambiae* mosquitoes in Liberia (Table 2) [37]. This low identity suggests that this is also a novel virus species, designated as Murici virus (MURV).

**Fig 4:**
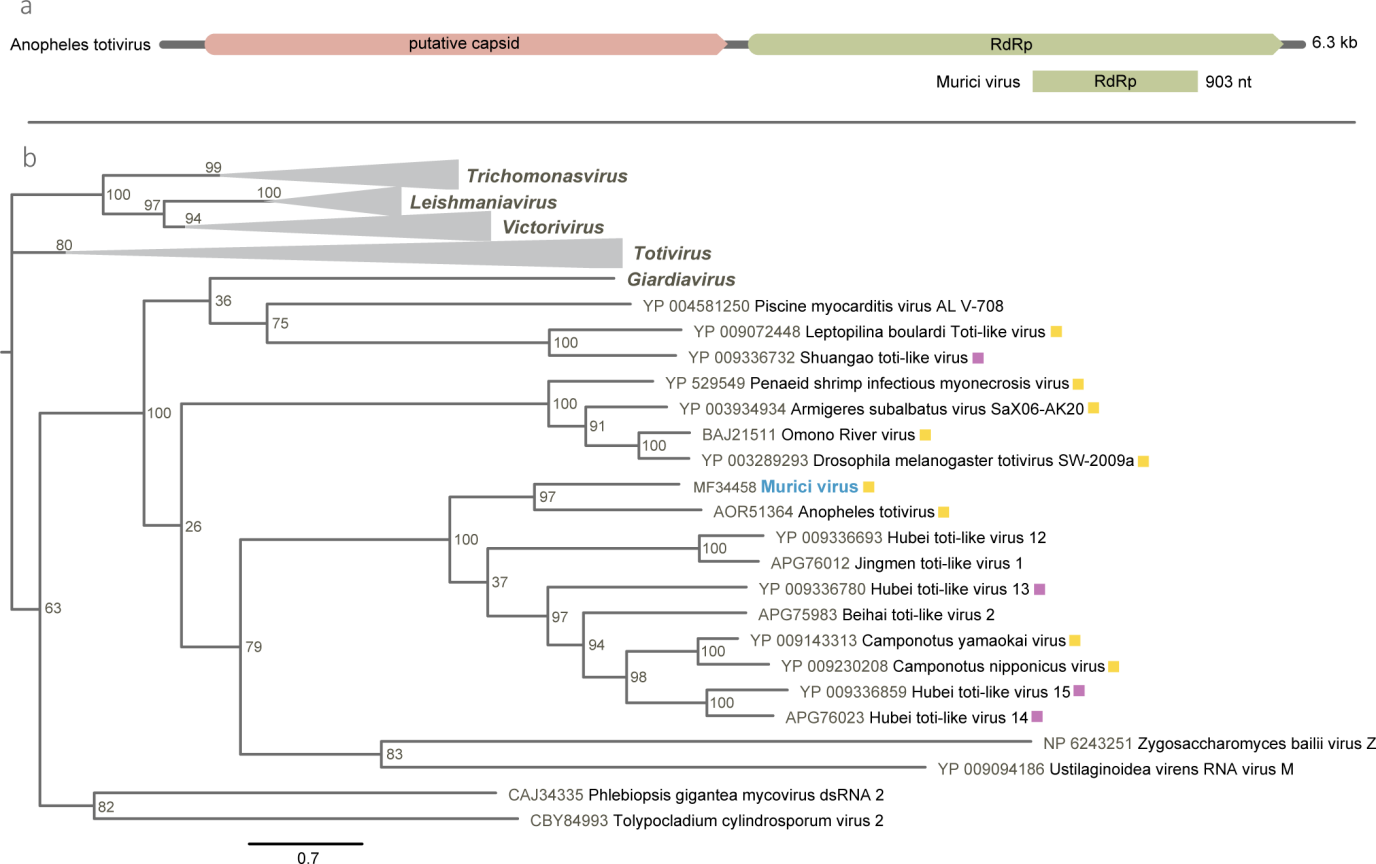
Murici virus genomic map and phylogeny. (**a**) RNA dependent RNA polymerase (RdRp) protein of Murici virus and Anopheles totivirus. (**b**) Maximum likelihood phylogenetic tree for RdRp sequence of Murici virus (in blue) with respective most related members of *Totiviridae* family. Viruses originally found in arthropods and other insects are marked in yellow and purple, respectively. Bar indicates amino acid substitutions per site.

The ML phylogeny based on the RdRp with representative members of the *Totiviridae* family related to MURV grouped this virus with AToV in a separated clade with high node value, clustered within a major group that include unclassified arthropod viruses. The five *Totiviridae* genera are originally arranged in three initial groups, where the unclassified virus set is closer to the Giardia lamblia virus isolate Wang, the only member of G*iardiavirus* genus (Fig 4).

### Partitiviridae

Two putative RdRp partiti-like sequences encoding 456 and 381 aas were detected in the pools of salivary glands of *Ma*. *wilsoni* (M05) and *Culex* sp. (M06), related to the Hubei partiti-like virus 42 and the Hubei partit-like virus 48 with 56 and 57% identity, respectively. These divergent sequences of a highly conserved genomic region indicate the presence of two new virus species different between each other, named as Araticum virus (ARAV) and Angico virus (AGIV) (Table 2).

**Fig 5:**
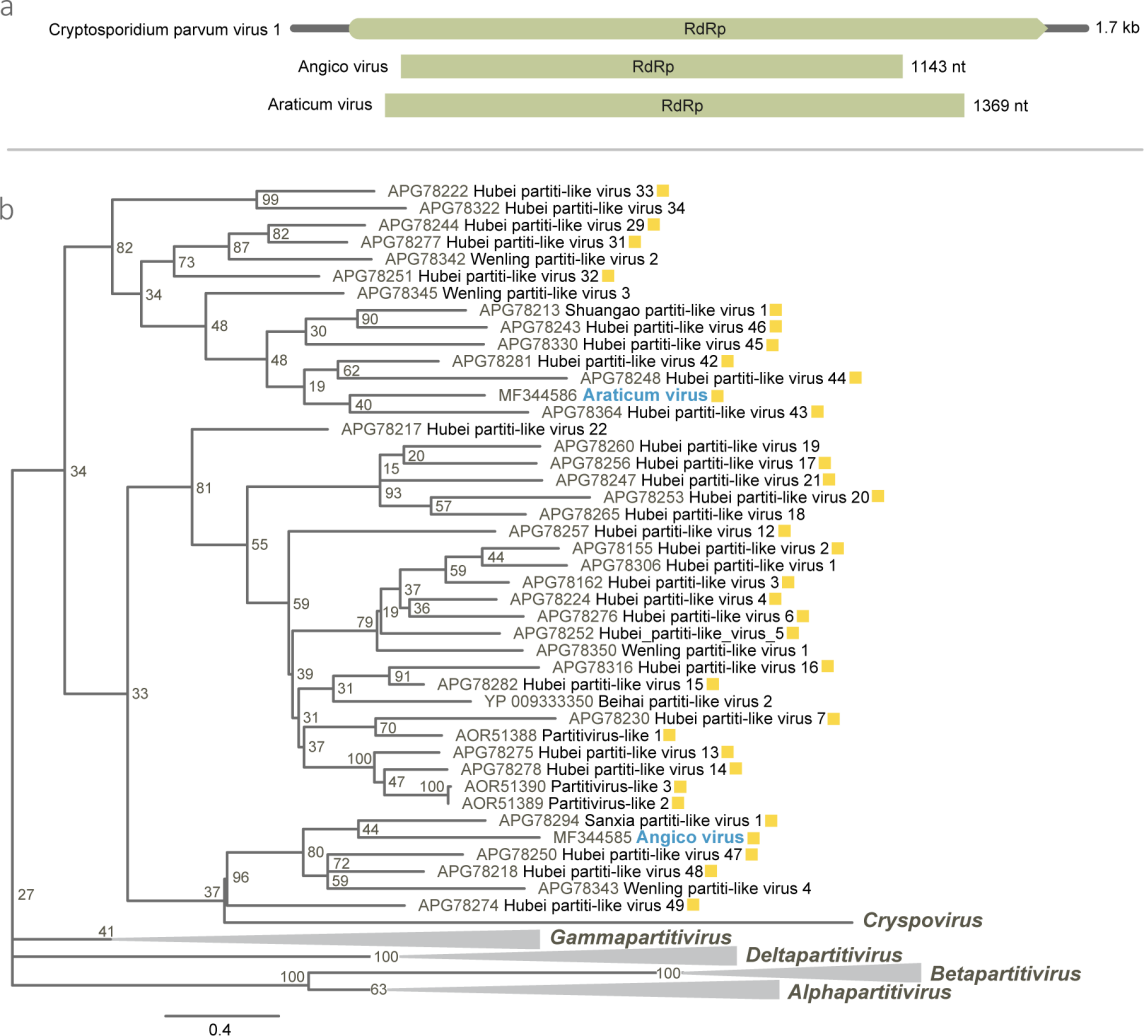
Genome map of Angico and Araticum viruses and phylogeny. (**a**) RNA dependant RNA polymerase (RdRp) gene comparison of Angico and Araticum viruses and Crypstosporidium parvum virus 1. (**b**) RdRp Maximum likelihood phylogenetic tree of Angico virus and Araticum virus (in blue) and other *Partitiviridae* members. Viruses originally found in arthropods are marked in yellow. Bar indicates amino acid substitutions per site.

The AGIV and ARAV ML tree was based on all approved members of the *Partitiviridae* family RdRp sequences. A large group of recently discovered viruses includes AGIV and ARAV and stands distinctly although with a common ancestor to four other *Partitiviridae* genera. This entire group behaves as a distinct lineage of Cryptosporidium parvum virus 1, the unique member of the *Cryspovirus* genus, comprised by several arthropod viruses described in a study carried out in China (Fig 5) [8].

## Discussion

Metagenomic studies contribute to the discovery of a great number of new viral species worldwide [3,8,38]. In this study, the sequencing of viral RNA obtained from the salivary glands of 66 Culicinae females collected in Chapada dos Guimarães National Park demonstrated the presence of previously undescribed ISV. These viruses belong to the *Chuviridae*, *Rhabdoviridae*, *Partitiviridae* and *Totiviridae* families, all comprising RNA viruses, clustered with other arthropod viruses recently described within these families.

Rhabdoviruses are ssRNA- viruses pathogenic to humans, animals and plants, including also a large number of unassigned viruses associated with a wide array of insects and other arthropod species with global distribution [16,39,40].

The LOBV genome detected in this work contains the general layout found in rhabdoviruses, flanked by five structural protein genes in the order 3’-N-P-M-G-L-5’, clustered together in a monophyletic group with three rhabdoviruses, RISV, Tongilchon virus 1 and NOCRV. This group composes clade I of the recently proposed *Dielmovirus* [41], a new genus from *Rhabdoviridae*, which was also formed for another set of viruses, clade II, and behave as a basally rooted lineage for the dimarhabdovirus supergroup (Fig 2). At the present, the dielmoviruses described were identified in mosquitoes from Australia (NOCRV and Beaumont virus) [34], Hungary (RISV) [35], Japan (Culex tritaeniorhynchus rhabdovirus) [42], Mexico (Merida virus) [43] and South Korea (Tongilchon virus 1) [44].

KAIV was recently discovered in *Culex* mosquitoes from the South-Pantanal region of Mato Grosso do Sul State, Brazil, closely related to Guato virus (GUTV) with 71% aa identity [33]. Our data suggest that these viruses, as well as CUMV and CROV, are Brazilian members of the *Chuviridae* family. This family was proposed for a large monophyletic group of newly discovered RNA viruses presenting distinct genome organization, including unsegmented, bi-segmented and a circular form of ssRNA-, that behaves phylogenetically as an older divergent group of rhabdoviruses [16].

GUTV and the original KAIV sequences only encode an incomplete putative glycoprotein, as well as all chuvirus-like sequences found in four different pools of this study. Finding the complementation of these genomes can be difficult, since glycoprotein may be so diverse that the available search tools are unable to map their contigs with known viral proteins, making the discovery of very distinct viruses a challenge. Additionally, KAIV was found in the salivary glands of *St*. *albopicta*, different from the original description in *Culex* spp., indicating that this virus infects different species of Culicinae.

Some ISV belonging to the *Bunyaviridae*, *Flaviviridae* and *Rhabdoviridae* families are ancient RNA viruses [21] with highly divergent lineages, indicating that their evolution accompanied the evolution of their respective hosts [45,46]. Integration of these viruses into mosquitoes genomes [47–49] and their adaptation to vertebrates and plants is widely proposed as the probable origin of pathogenic viruses for these hosts [16,50].

The totivirus Murici virus (MURV) detected in this study infecting *Ma*. *wilsoni* mosquitoes is closely related to Anopheles totivirus, found in *Anopheles gambiae* mosquitoes in Liberia [37], being tentatively classified within arthropods viruses of *Artivirus* genus belonging to the *Totiviridae* family. *Totiviridae* members commonly have a monossegmented dsRNA genome, organized in two overlapping ORFs, which encode the major capsid protein and the RdRp. These viruses are originally known to infect protozoa and fungi of importance for humans, animals and plants [51]. However, several arthropod totiviruses have also been frequently found lately and the *Artivirus* genus (arthropod totiviruses) was proposed to classify them within the family [37,52,53].

The *Partitiviridae* family was recently reorganized and beyond the *Cryspovirus* genus (protozoa viruses), four new genera were included: *Alphapartitivirus* and *Betapartitivirus* (fungi and plant viruses), *Gammapartitivirus* (fungi viruses) and *Deltapartitivirus* (plant viruses) [54]. The ML tree for ARAV and AGIV supports the need to create a new group for the current unclassified viruses of this family, more closely related to the genus *Cryspovirus*. *Partitiviridae* members present bi-segmented dsRNA genomes, typically associated with latent infections in a wide range of fungi, plants and protozoa [54]. Although unlikely, the totivirus (MURV) and partitiviruses (ARAV and AGIV) found in this study may represent new species of viruses from microorganisms and parasites, rather than ISV.

Some investigations with dual infection in mosquito cells or live mosquitoes demonstrated that ISV isolated from Culicinae mosquitoes such as Palm Creek virus, Nhumirim, Culex flavivirus and Bagaza can reduce the replication of certain arboviruses when previously inoculated, such as the West Nile, Murray Valley encephalitis, Japanese encephalitis and Saint Louis encephalitis viruses [55–60]. Despite the possibility of using this ability as a control of important public health arboviruses present in vector populations, little is known about the real influence of ISV on mosquito competence and, therefore, on the transmission of arboviruses to humans [15,21,58].

Finally, it is possible to verify that our findings correlate to newly described and very diverse viruses, reinforcing a stair climbing profile for viral diversity studies, where the current new viruses act as a necessary step in the discovery of future new viruses. Thus, our data contributes directly to better understanding viral salivary gland diversity in wild-type Culicinae mosquitoes, allowing the most precise and complete description of these viral families, as well as new alternatives for further studies on the viral symbiotic interference in mosquito vector competence for viruses with medical importance to humans, animals, or plants.
